# Supplementary figures and images for: Evolutionarily conserved brainstem architecture enables gravity-guided vertical navigation
Source: PLoS Biol. 2024 Nov 12;22(11):e3002902. doi: 10.1371/journal.pbio.3002902 (PMC11584107; doi:10.1371/journal.pbio.3002902)

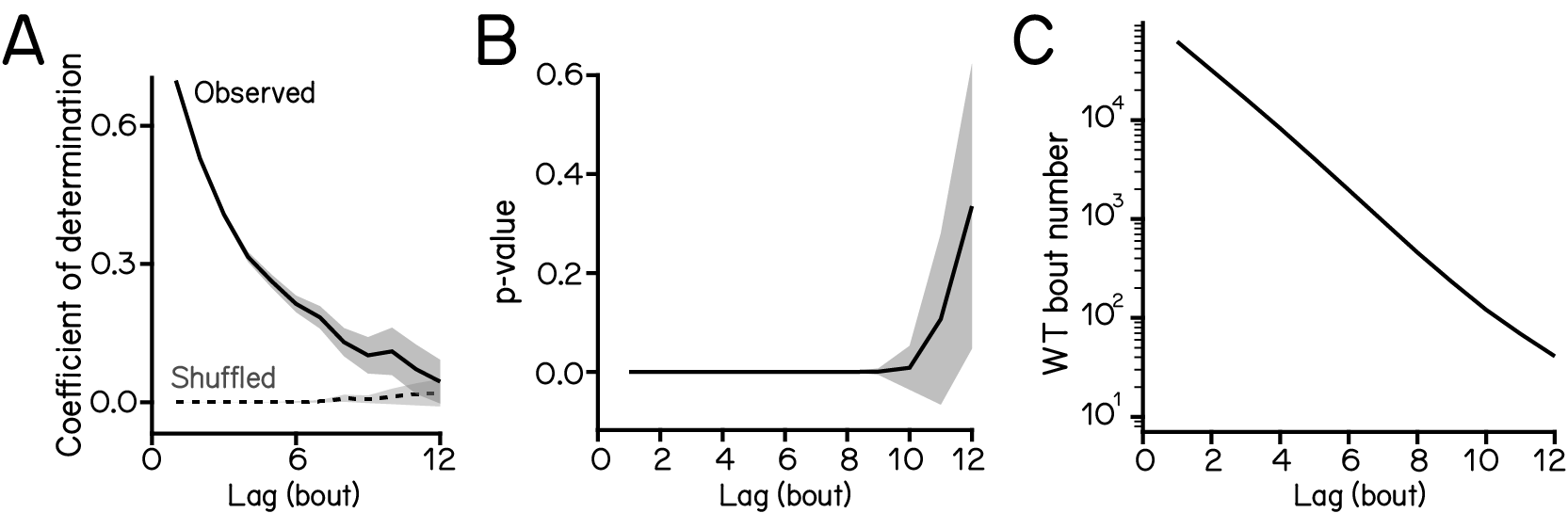

Supplement: S1 Fig — (A) Coefficient of determination plotted as a function of lag, extended to 12 bouts in a series. Values are calculated using all bouts. Standard deviations of bootstrapped data shown as shaded errors. (B) Correlation p value plotted as a function of lag. Values are calculated using all bouts. Errors indicate standard deviations of bootstrapped results. (C) Number of recorded bout sequences plotted as the length of the bout sequence. All code and data can be found at DOI: 10.17605/OSF.IO/AER9F. (TIF) [file pbio.3002902.s001.tif]

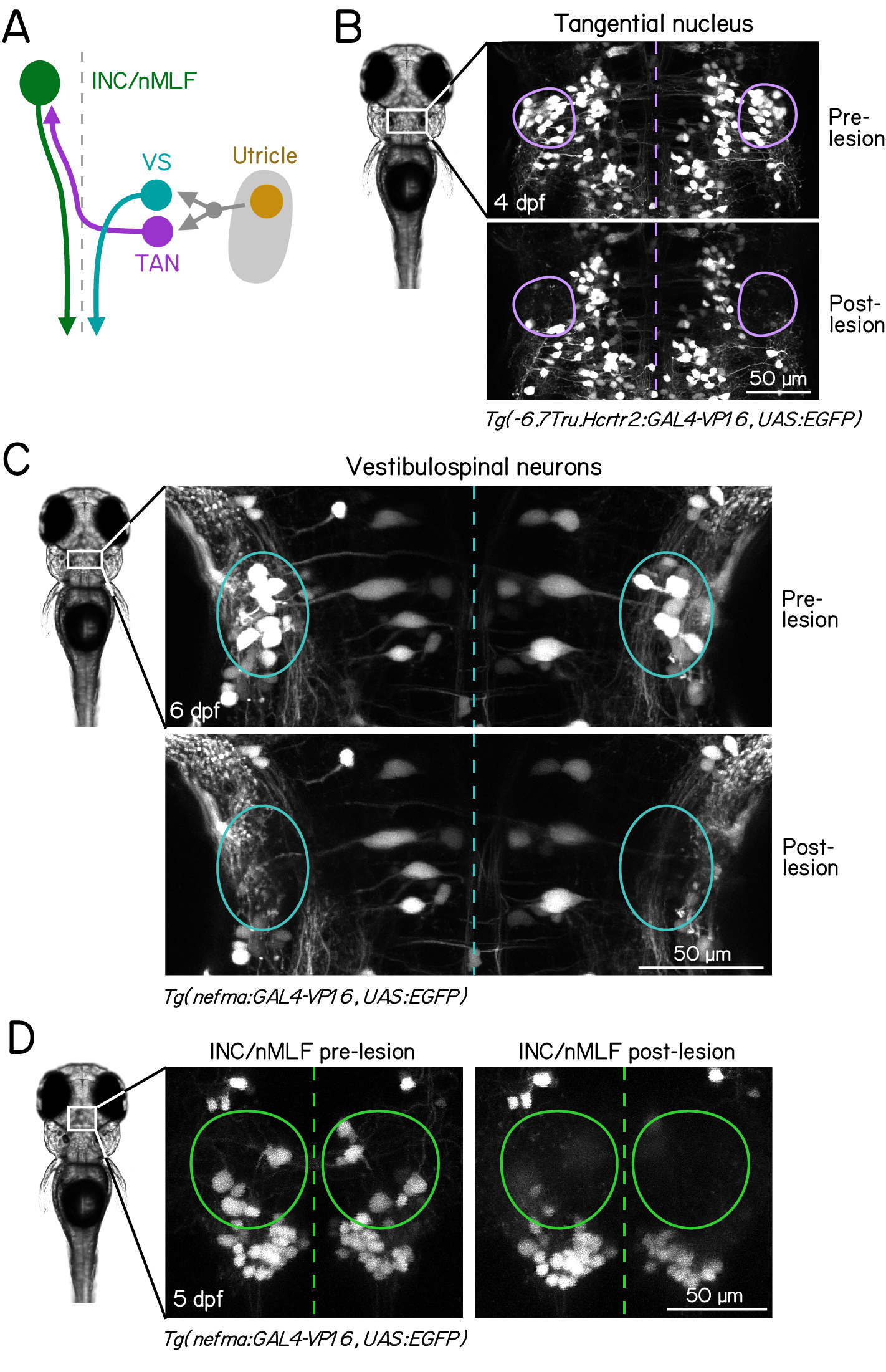

Supplement: S2 Fig — (A) Schematic diagram of vestibular circuits in the brain-stem. Utricle: utricular otoliths (yellow); TAN: the tangential vestibular nucleus (magenta); VS: vestibulospinal neurons (cyan); INC/nMLF: the interstitial nucleus of Cajal/the nucleus of the medial longitudinal fasciculus (green). (B) Before and after lesions of the tangential vestibular nucleus (circled) in a 4 dpf larvae. Scale bar: 50 μm. (C) Before and after lesions of the vestibulospinal nucleus (circled) in a 6 dpf larvae. Scale bar: 50 μm. (D) Before and after lesions of large neurons in the INC/nMLF (circled) in a 5 dpf larvae. Scale bar: 50 μm. All imaging data can be found at DOI: 10.17605/OSF.IO/AER9F. (TIF) [file pbio.3002902.s002.tif]

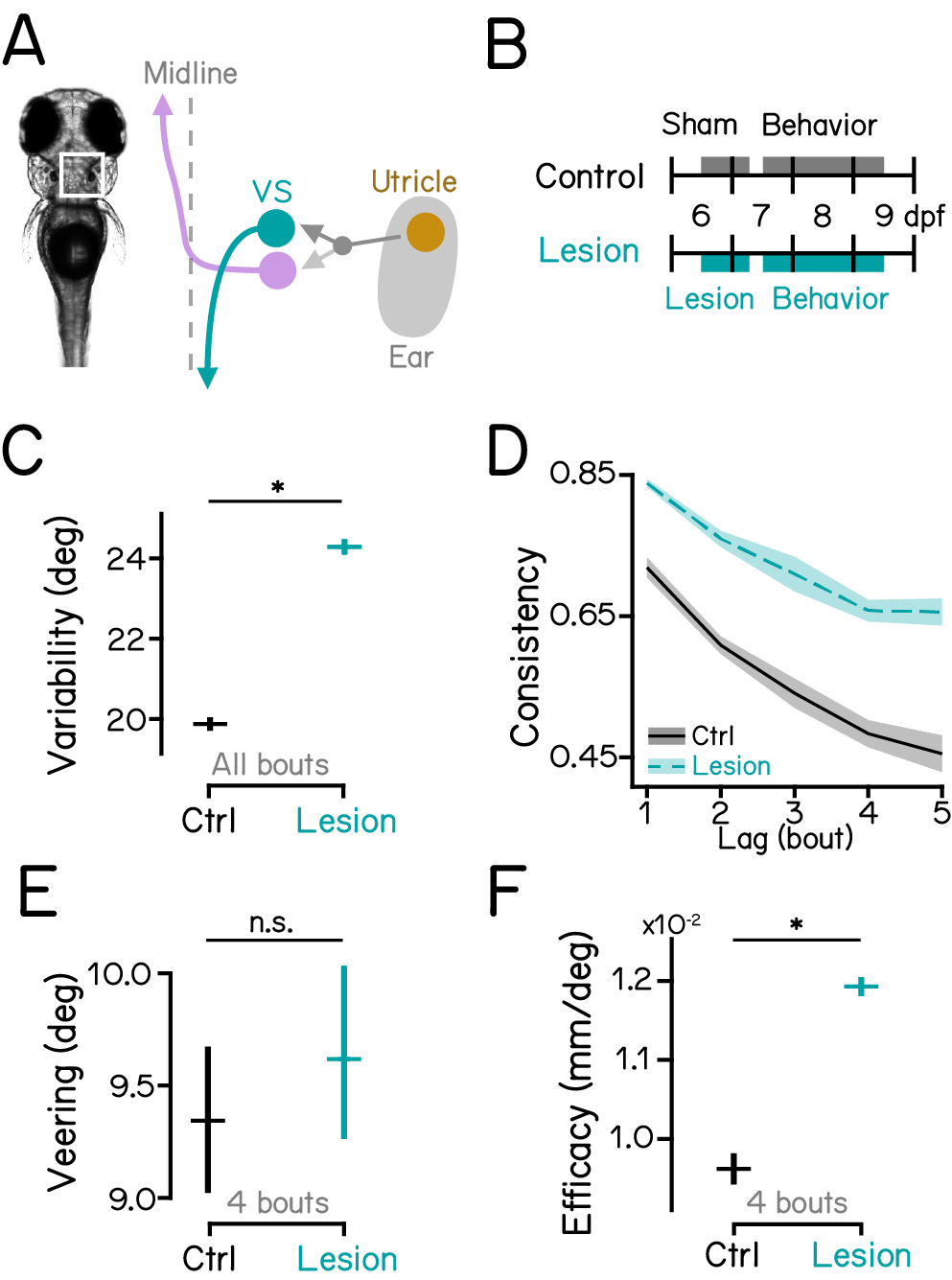

Supplement: S3 Fig — (A) Schematic view of the inner-ear utricular otolith and the vestibular pathways in the hindbrain of zebrafish. Utricle: utricular otoliths (yellow); VS: vestibulospinal neurons (cyan). (B) Diagrams of experimental procedures for lesions of the vestibulospinal nucleus and behavioral assays. See S2 Fig for examples of lesions. (C) Swim direction variability compared between vestibulospinal-lesioned larvae and controls. The means of boot-strapped MADs are plotted with error bars showing standard deviations. n = 18,366/18,363 bouts from 79/97 fish over 8 repeats for controls/lesions. Pbootstrap = 1.42e-60. (D) Swim direction consistency plotted as a function of the number of bouts in the sequence. Shaded bands indicate standard deviations of the slope estimated using bootstrapping. (E) Veering through 4 consecutive bouts plotted in median with 95% confidence intervals. n = 3,554/2,920 4-bout series from 79/97 fish over 8 repeats for controls/lesions. Pmedian-test = 0.154. (F) Depth change efficacy plotted as bootstrapped means with error bars showing standard deviations. Pbootstrap = 1.61e-24. See also Tables 2 and 3 for statistics. All code and data can be found at DOI: 10.17605/OSF.IO/AER9F. (TIF) [file pbio.3002902.s003.tif]

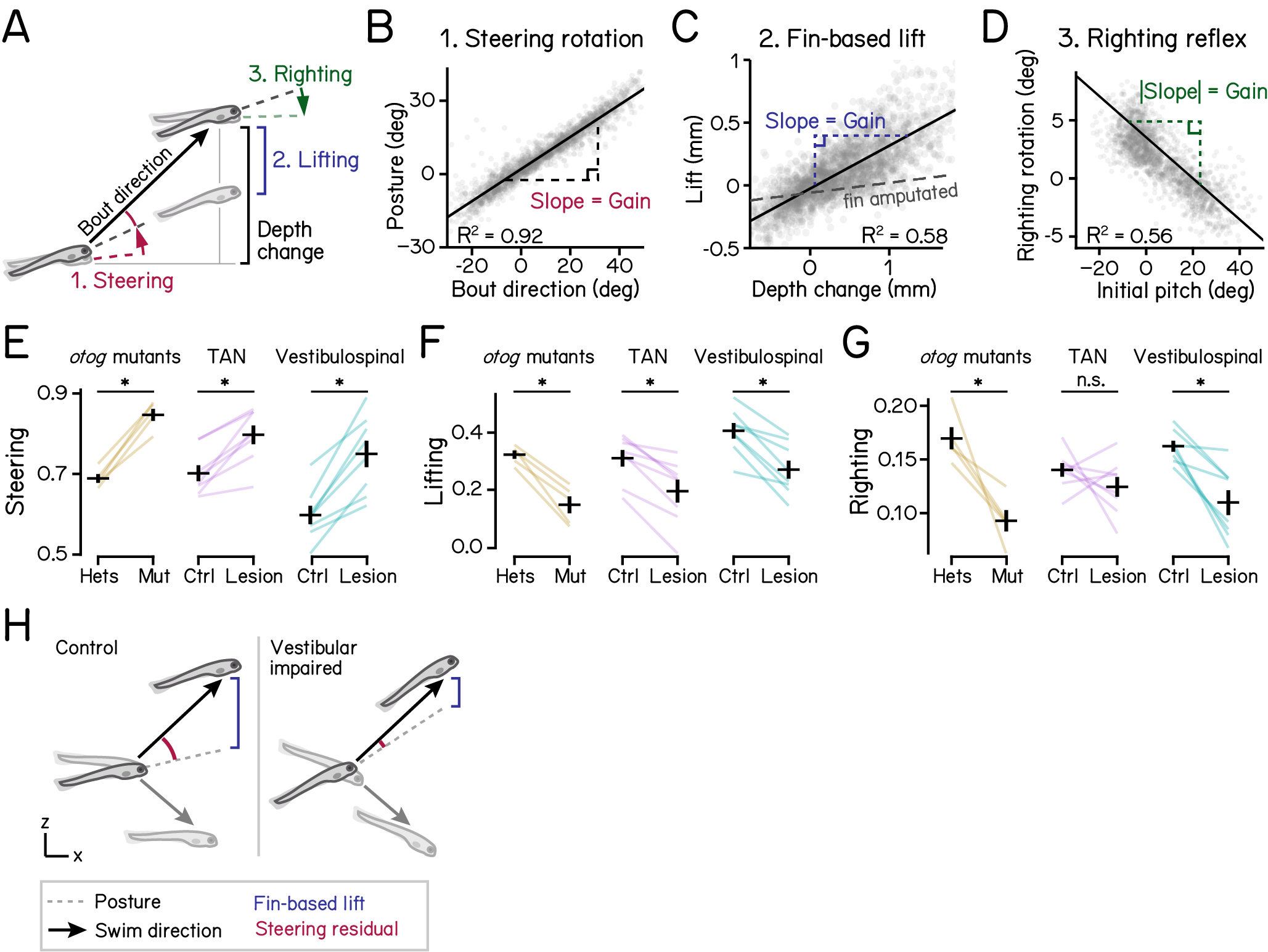

Supplement: S4 Fig — (A) Schematic diagram showing steering, lifting, and righting during a swim bout. Larvae steer toward targeted direction during acceleration (red arrow), use pectoral fins to assist in depth changes (blue), and restore posture to horizontal during deceleration (green arrow). Z displacement generated by lifting (blue) is estimated by subtracting theoretical displacement in depth, calculated from the head direction and x distance, from the total depth change. (B) Steering gain is defined as the slope of the best fit line of posture at the time of the peak speed vs. swim direction. n = 121,979 bouts from 537 fish. (C) Lift gain is defined as the slope of the best fit line of estimated lift vs. depth change of the swim bout. Pectoral-fin amputation reduces lift (dashed line). n = 33,491/28,604 bouts from 74/78 fish for control/fin-amputated. (D) Righting gain is defined as the numeric inversion of the slope of the best fit line of rotation during deceleration vs. initial posture. n = 121,979 bouts from 537 fish. (E) Steering gain of vestibular-impaired larvae vs. controls. otog mutation: Pt-test = 2.33e-5; tangential lesions: Pt-test = 7.41e-3; vestibulospinal lesions: Pt-test = 2.32e-3. N = 5/8/8 experimental repeats for otog/tangential lesions/vestibulospinal lesions. Same as follows. (F) Lifting gain of vestibular-impaired larvae vs. controls. otog mutation: Pt-test = 7.09e-4; tangential lesions: Pt-test = 3.57e-2; vestibulospinal lesions: Pt-test = 5.90e-3. (G) Righting gain of vestibular-impaired larvae vs. controls. otog mutation: Pt-test = 6.76e-4; tangential lesions: Pt-test = 0.180; vestibulospinal lesions: Pt-test = 1.00e-3. (H) Summary of effects of vestibular perturbations on bout kinematics. Vestibular-impaired fish swim with more eccentric posture and less fin-based lift. TAN, tangential. See also Table 1 for parameter definitions and Table 3 for statistics. All code and data can be found at DOI: 10.17605/OSF.IO/AER9F. (TIF) [file pbio.3002902.s004.tif]
