# Supplementary material for: Evolutionarily conserved brainstem architecture enables gravity-guided vertical navigation
Source: PLoS Biol. 2024 Nov 12;22(11):e3002902. doi: 10.1371/journal.pbio.3002902 (PMC11584107; doi:10.1371/journal.pbio.3002902)
Supplement: Attachment — Submitted filename: R2RGN.pdf [file pbio.3002902.s005.pdf]

August 12, 2024

We are grateful for the Reviewers' thoughtful and interesting comments and questions. In response we have made substantial revisions allowing for a clearer and stronger manuscript. Below please find a point-by-point response to each of their comments.

Reviewer #1

The paper "A brainstem circuit for gravity-guided vertical navigation" by Zhu et al., investigates how larval zebrafish structure navigational movements in the depth direction. In larval zebrafish, turn directions and swim-speeds in the horizontal plane are structured rather than selected at random during both spontaneous exploration as well as navigation. While navigational strategies in the horizontal plane have been examined in many animal species, if and how movements are structured in the "z-direction" is far less explored. Given the importance of navigation in height or depth across species, this manuscript begins to address the critical question of how z-movements may be structured in order to allow for efficient exploration or navigation in depth. I strongly believe that this will be of great interest to the scientific community. The paper is very clear, well laid out and the methods used are highly appropriate for the study. I truly enjoyed reading it.
While I was initially unsure whether the word "navigation" is appropriate in the title of the paper, considering that there does not seem to be a clear goal presented to the zebrafish, I do agree with the authors that a) navigation can be understood broadly as a means to accomplish movement in a particular direction and b) that the presented data strongly argues that the building blocks identified by the authors serve goal directed navigation as well. While I think that this manuscript should be published, I have one major reservation that has to be addressed.

We thank the reviewer for the kind comments, recognition of the significance of our findings, and perspective on "navigation."

It is unclear what data the reported statistics are based on. Table 2 suggests that the statistics compare across fish (i.e. N=<number of fish>), however, the figure legends and methods are very ambiguous about this point. If statistics are indeed calculated across fish averages, this should be clarified in the figure legends (by stating the relevant N rather than both the number of fish and the number of swim bouts) and made explicit in the methods. If statistics are currently calculated across individual swim bouts, this should be corrected. Given that the manipulations carried out / used by the authors (ablations, mutations) are performed on a per-fish and not on a per-bout basis, the natural N would be the number of fish tested.

We apologize for the confusion. To achieve the necessary spatial and temporal resolution, our behavioral apparatus has a limited field of view, and only measures a subset of bouts made by an individual fish. Empirically, if we put a single fish in the apparatus, we observe too few bouts to estimate navigation parameters. Instead, we put multiple fish in each apparatus simultaneously and so cannot analyze data on a per-fish basis.

For wild-type data, each biological replicate, or "experimental repeat" consists of all bouts produced by siblings from a single clutch that were run on a given day. Different clutches of animals, on different days, were treated as independent. As wild-type data is straightforward to collect and abundant, each "experimental repeat" consists of sufficient bouts to stably estimate each navigation parameter. The "N" is therefore simply the number of experimental repeats (22). We have added this number to the figure legend where appropriate.

For manipulations, an "experimental repeat" similarly consists of siblings run as "control" and "perturbed" on a given day. Lesioning a single fish takes considerable time, and so a single "experimental repeat" generates an order of magnitude fewer bouts than a wild-type "experimental repeat." To evaluate whether our perturbation disturbs navigation, we 1) pool data across repeats 2)

bootstrap to generate 100-500 estimates of the expected value for the control and perturbed data sets and 3) generate a test statistic and p value by comparing these estimates (Table 2); since the p value is computed explicitly for such permutation testing, and not estimated with respect to a theoretical probability distribution, we do not report an "N," and have left the total numbers of bouts and fish in the figure legend. Since we could estimate locomotor parameters for single "experimental repeats," we did so, reporting the N (5-8) in Table 3 and in the legend for Figure S4.

To avoid confusion going forward, we 1) added the paragraphs above to the Methods section under "Statistics" and 2) added additional detail about the number of "experimental repeats," bouts, and fish in figure legends and Tables 2&3. In addition, we removed dots/datapoints representing values of bootstrapped navigation parameters in Figures 2-4 and S2 (now S3), and maintained lines indicating locomotor parameters calculated from individual experimental repeats in Figure S3 (now S4). We hope that this makes our experimental and statistical approaches clearer.

I would be curious to know at what point the correlation of consecutive bouts reaches chance level, i.e. becomes indistinguishable from shuffled controls. I understand that there might be a limitation of SAMPL in how long trajectories it can capture, however if it is possible to get at this point, I think it would make a worthwhile addition. Compared to angular correlations observed in the horizontal plane, these R^2 values are strikingly high, and knowing when they reach chance level could give a bound on streak length used during navigational movements.

We explored this question using the wild-type dataset and found that the direction of the 11th bout (lag = 11) is indistinguishable from shuffled data (Figure R1 A). As expected, the number of data points drops as lag gets longer (Figure R1 C). Consequentially, the correlation p-value increases rapidly as we look beyond the 9th bout (lag = 9) (Figure R1 B), and so to be conservative, our best answer is that consecutive bouts reach chance level between the 9th-11th bout. In the revised manuscript, we included this this figure (Figure R1) as a supplemental figure in the revised manuscript (Supplemental Figure 1). Finally, we agree that the high R^2 is noteworthy; it gives us confidence in our proposal that larvae truly do navigate in depth.

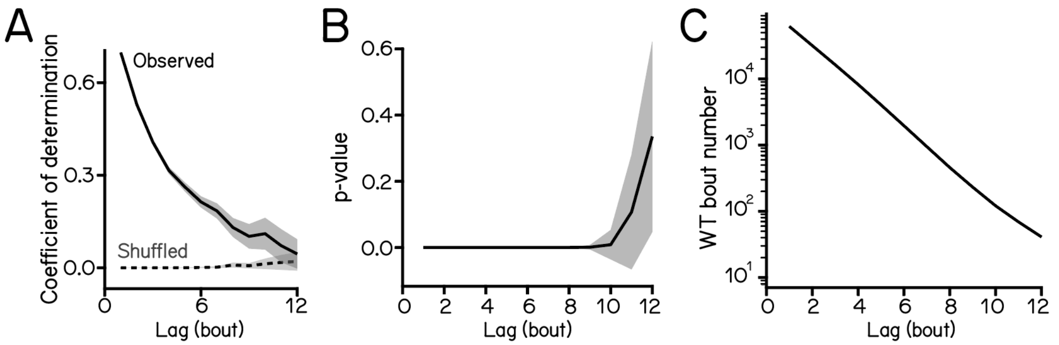

Figure R1.
(A) Coefficient of determination plotted as a function of lag, extended to 12 bouts in a series. Standard deviations of bootstrapped data shown as shaded errors.
(B) Correlation p-value plotted as a function of lag. Errors indicate standard deviations of bootstrapped results.
(C) Number of recoded bout series with given length of the bout series.

While I'm generally fine with the use of the word "navigation" (see above), I do think that speaking of vertical navigation efficiency (first paragraph of the Discussion) is a bit misleading. Mainly, if there were a goal, maintaining consistent upward or downward trajectories once the goal has been reached would actually be highly inefficient as a navigational strategy.

On reflection, we agree with the Reviewer that, without a clear goal, “efficiency” is not the best term. In the revised manuscript, we softened our statement and used depth change efficacy to describe this behavior (Figure 1L-O, and first paragraph of the Discussion). To rule out the effect of swim distances on depth changes, we quantified ratio of the depth change and the swim distance (Figure 1L-O). Given the swim direction of a bout, a fish with higher depth change efficacy translocates more in the vertical axis following consecutive bouts in the sequence (Figure 1L). We therefore think this is a better measurement of the efficacy of vertical navigation and are grateful to the Reviewer for this push. One final note: as the consistency measures are smaller than 1, the trajectories of climbs and dives gradually approaches horizontal. We think this allows fish to avoid overshooting. Instead, they appear to reach a goal in depth and end the climbs/dives with a stable and horizontal bout.

Reviewer #2

The manuscript of Yunlu Zhu et al. titled "A brainstem circuit for gravity-guided vertical navigation" describes a circuit involved in navigation based on the perception of gravity. They use genetic and targeted manipulation to explore the role of a conserved brainstem circuit in the sensorimotor transformations involved in vertical navigation in zebrafish.

Overall, the manuscript is clear and well written, as are the figures. The data is well presented and easy to understand and follow. I am however concerned by the mildness of their manipulations' effect sizes, and the fact the data does not appear to support their conclusions. Despite mentions of crucial, indispensable, or essential, the data represents changes of 5-10 % in navigation efficiency.
The experimental design is appropriate, and the statistical analysis correct. The tables recapitulate all the data very nicely and conveniently. The code provided seems adequate, although I have not tested it. The supplementary information are welcome, and the methods detailed. However, I do not think the results are provocative, or would have a general interest to a wider audience. The very strong causal language used throughout the manuscript is not supported by the data in my opinion, which is detrimental to the manuscript as a whole.

We struggled to square Reviewer 2’s many thoughtful insights and helpful critiques with their characterization of the results and potential interest. We take the point that the manuscript would be stronger if the claims were tempered and have done so throughout the manuscript. We’ve also done our best to explain why small changes in efficacy have profound consequences for navigation in our responses below and in the updated manuscript. We remain grateful for the Reviewer’s continued engagement and hope our updated manuscript meets their high bar.

There seems to be a high variability between experiments, and it is unclear if each comes from a different clutch? For example, the vestibulospinal neurons controls (S2) are worse than many of the manipulations in the other parts of the paper.

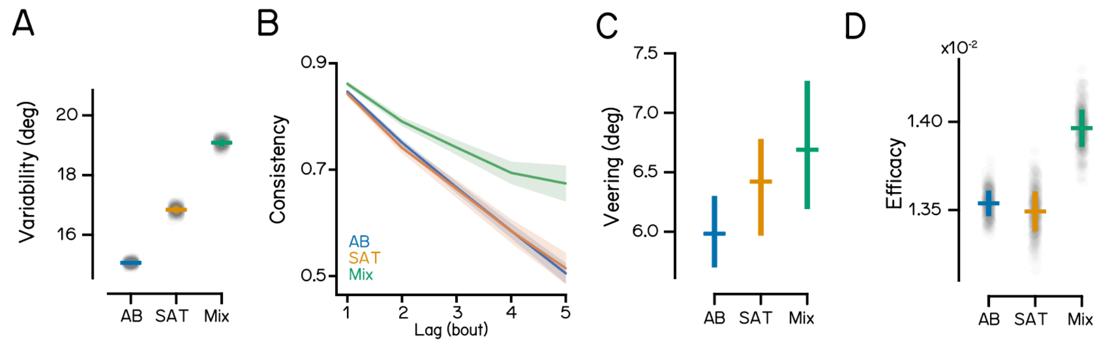

Figure R2.
(A-D) Navigation parameters of wild-type larvae (cousins) with different background strains.
Larvae were generated by crossing a line of transgenic fish to wild types in 3 corresponding backgrounds.

Yes, across experiments the fish are from different clutches: the experiments presented represent data gathered over the course of six years from larvae on different genetic backgrounds. We've previously reported that genetic background can impact locomotor parameters (Zhu et al., 2023, https://doi.org/10.1016/j.celrep.2023.112573). To provide some context, we've repeated the analyses in the manuscript on the datasets from that paper (Figure R2) As expected, we can see variation in the navigation parameters we analyze here among the three different strains of zebrafish. We therefore believe that background is responsible for most of the variability between different control groups. Since all of the comparisons we perform in the paper are between lesioned fish and their un-lesioned siblings, and not across perturbations, we believe our results stand. In the revised manuscript we now discuss genetic background in the "Limitations" section of the Discussion.

The effect of the otog-/- and lack of utricle is surprisingly small (less than 5%) in 2B.
A figure similar to 1K for the mutants would help assess their trajectory.

We now included a panel for the trajectories of the otog-/- mutants in Figure 2B. We are grateful for this suggestion, as it allowed us to clearly illustrates the consequences of the small change to variability on navigation.

We've puzzled over the Reviewer's claim that the small effect is surprising. Our best guess is that the Reviewer's surprise reflects confusion about what the "variability" reflects. We note first that "variability" does *not* reflect orientation. In the dark, *otog -/-* larvae are observed to swim at all orientations relative to gravity: upside-down, sideways, etc.. Consequentially, the variance in the distribution of *orientations* adopted by *otog -/-* fish is far far greater than wild-type siblings. Instead, "variability" is a measure that describes trajectory. Bout trajectory is a function of how much a fish rotates in the pitch axis. Losing the utricular otolith does not change anything about the motor control circuits or muscles that perform those rotations, nor does it change the morphology of the fish. Therefore, we were not surprised to see a mild increase in variability. One comparable data set that might be of interest to the Reviewer are the changes seen in patients with vestibulopathy. There, patients show small but significant, changes to gait variability (Grouvel et al., 2024 https://doi.org/10.1038/s41598-024-62335-1). Similar logic applies: a sensory deficit does not change the capacity of the motor system, only the ability to regulate it appropriately. We hope this additional context resolves the Reviewer's surprise.

Furthermore, it seems that the consistency of the WT in 1G is intermediary between the Hets and the Mut in 2C (which is confirmed by the very useful Table 1 and 2). The same with the efficacy, which is only 9.91e-2 for the WT in Table 1, compared to 9.05e-2 for the mutant. This value was deemed efficient on line 69, so it seems unclear how important the utricle truly is based on those results and how this data supports the "crucial" role of this otoliths in the observed behaviour.

As discussed above, we feel uncomfortable evaluating our data in the way the Reviewer does here because it conflates data from fish with different genetic backgrounds. We nonetheless take the Reviewer's point, have removed the word "crucial" from the manuscript and tempered our claims.

What is the explanation for the increase in efficiency of the vestibulospinal-lesioned larvae?

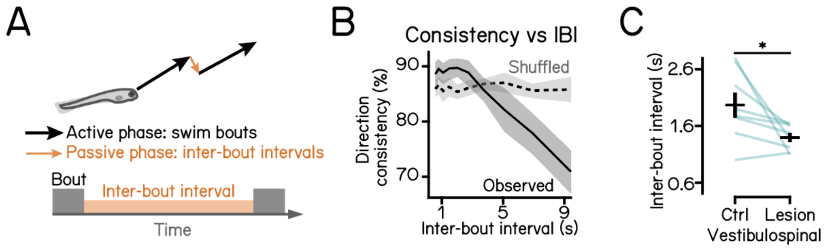

Figure R3.
(A) Schematic ilustration of an inter-bout interval.
(B) Direction consistency plotted as a function of inter-bout interval duration.
(C) Quantification of inter-bout invervals of vestibulospinal lesioned fish.

We share the Reviewer's curiosity, particularly since we've observed similar increases after other non-lesion manipulations of vestibulospinal circuits in the lab. Our current hypothesis is that the increase reflects a change in movement timing. The longer larvae wait between swim bouts, the less consistent the direction of their swims will be (Figure R3). Vestibulospinal-lesioned larvae have shorter inter-bout intervals (i.e. they swim more frequently) which means that torques due to gravity that rotate the fish have less time to act before they are corrected by a swim (c.f. Ehrlich & Schoppik 2017). That said, we have shown that fish determine when to swim based on other factors such as their posture and rotation (Figure 2 in Hamling et al., 2024 doi:10.1523/JNEUROSCI.2315-23.2024). Loss of vestibulospinal neurons might interfere with the sensitivity to posture / rotation, which might be the underlying reason for the difference in movement timing. In the revised manuscript, we included inter-bout interval measurements in Table 3 and discussed the increased efficacy in VS lesions.

The observations of swim kinematics are interesting, consistent, and raise a lot of questions. It seems that the larvae with any manipulation have a defect in their posture and orientation in the gravity field. But at that stage of development, it is unlikely that the semicircular canals are functional. How would the swim kinematics be affected? I find the results of 3H difficult to reconcile with the opposite effects of TAN and Vestibulospinal lesions on vertical navigation (3C and S2C). This is discussed (161-167), but there is no speculation or attempt at a conclusion that would reconcile these observations.

We were initially unsure of whether to include the measurements of swim kinematics and are gratified to infer that they were provocative and interesting to the Reviewer. At the ages we have studied, the Reviewer is correct that the semicircular canals do not respond to the rotations produced during swimming. We are unclear as to why the semicircular canals would play a role here; as the Reviewer no doubt knows, the canals act as rotational, not linear accelerometers. Interfering with the primary gravity-sensing organ (the utricle) or downstream circuitry is what we expect would produce defects in posture and orientation in the gravity field.

Previous work in our lab has parameterized and modeled the kinematics shown in Figure S2 (now S3 in the revised manuscript). Briefly, larvae coordinate body rotation, fin propulsion, and body undulation to perform a swim bout. However, these effectors can be flexibly modulated. For example, larvae with their pectoral fins removed increase body rotation to compensate for the loss of lift forces. There, we showed that utricular sensation plays a key role in coordinating fin and body (Ehrlich & Schoppik 2019, https://doi.org/10.7554/eLife.45839). The many degrees of freedom available to achieve a particular orientation / trajectory mean that speculation and conclusions are challenging to support. That said, we understand the Reviewer's point. Addressing it will require extending our models of swim kinematics in the pitch axis to incorporate the key finding of this manuscript: that there is structure to swimming on order 10 bouts that larvae use to navigate. While such a reconciliation is beyond the scope of the manuscript, we have elaborated these ideas in the Discussion.

What could be the contribution of the swim bladder to those vertical navigation? Somato- and proprioception could explain how the fish still largely maintain vertical navigation without gravity perception. This is a short report, so I do not expect the authors to address this question, but having some hypotheses to follow on would be of interest.

We acknowledge that there are other sensory systems and organs available to the larvae, and that they might contribute to navigation; we discuss this below. First, though, we respectfully disagree with the Reviewer's claim that "fish still largely maintain vertical navigation without gravity perception." We believe that fish maintain *trajectory* without gravity perception. Maintaining trajectory can be done without any sensory information at all. Instead, this phenomena can be explained simply by the fact that fish do not move like Brownian particles; instead, their pitch rotations from bout to bout are considerably more constrained. Consequentially, if a fish swims with a particular trajectory, its next bout can only deviate from that trajectory by so much. Globally, this means that even gravity blind *otog* -/- fish maintain trajectory in the dark, as in the example trajectories above. In the paper, we use the fact that, when information about gravity is available, additional correlation across bouts emerges and permits effective changes in depth. As it is above and beyond what would be expected from inertia, we believe this additional correlation to be evidence of navigation.

A loss-of-function experiment that targets somatosensation / proprioception / swim bladder inflation like we did with *otog* -/- would reveal if these senses / organs contribute similarly to the correlational structure across bouts and its consequences. To the best of our knowledge, no clean reagents exist for these experiments. For example, targeting spinal cord proprioception neurons may allow investigating their roles in vertical navigation, but such ablations comprise locomotor activity (Picton et al., 2021 doi:10.1016/j.neuron.2021.01.018). Finally, because the height of our apparatus is on the order of centimeters, we expect that the swim bladder would not respond meaningfully to pressure differentials in depth.

Is there any effect of the manipulations on the hunting capabilities of the larvae? Would that affect their development over the 2 days of the experiment?

We have not assessed their ability to hunt directly but have measured body length, which is an excellent indicator of developmental age. We did not observe a significant difference between controls and the conditional groups (Figure R4, datapoints indicate experimental repeats).

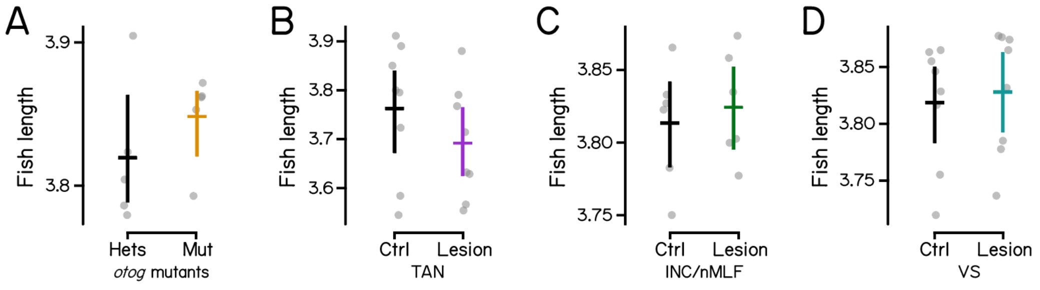

Figure R4.
(A-D) Fish length compared between control and conditional groups. Each dot represent one experimental repeat.

Minor comments
Line 161: First, it demonstrates…
Fixed

Figure S1: It may help to guide readers by adding the schematics showing the inner-ear and vestibular pathway and where each lesion is on that scheme.

We added a diagram showing the circuit pathway in Figure S2 in the revised manuscript (S1 in the original submission).

Reviewer #3

Zhu et. al. present clear data showing that the tangential nucleus and INC/nMLF constitute a circuit that receives vestibular input and plays a key role in vertical navigation. They establish an assay to observe vertical navigation in the water column, and measure important parameters of navigation, including consistency of bout direction and efficacy of depth change. The mutant and ablation data indicate that lesions to the tangential nucleus and INC/nMLF have effects similar to having no otolith, while vestibulospinal neuron ablations have a different phenotype. These findings lay important groundwork for further investigation into the circuits by which animals sense gravity and use it for navigation. However, I have a few comments that need to be addressed before publication:

We thank the reviewer for acknowledging the foundational importance of our work and for their thoughtful comments.

1. More explanation of why and how zebrafish larvae navigate in depth is needed in the Introduction. There was one sentence about this in the Discussion, but the authors should clarify earlier and more fully why it may be important for larvae to swim upwards and downwards.

We added examples where zebrafish change depth in the Introduction.

2. In plots of Variability and Efficacy, e.g. Figure 2 B and E, the legend says data points are plotted, but it looks more like a cloud, and it is impossible to see the individual points to get an idea of the distribution of the data. Some kind of density or violin plots would help to illustrate the range of individual fish values.

We agree that showing data on a per-fish basis would be helpful, but as discussed in the responses to Reviewer 1, the limits of our apparatus preclude measuring navigation parameters for individual fish. Briefly, to achieve the necessary spatial and temporal resolution, our behavioral apparatus has a limited field of view, and only measures a subset of bouts made by an individual fish. Empirically, if we put a single fish in the apparatus, we observe too few bouts to estimate navigation parameters. Instead, we put multiple fish in each apparatus simultaneously and so cannot analyze data on a per-fish basis. Further, lesioning a single fish takes considerable time, and so we 1) pool data across repeats 2) bootstrap to generate 100-500 estimates of the expected value for the control and perturbed data sets. Data in the figures references by the Reviewer represent the bootstrapped estimates of expected value. As there is no information about individual fish or experiments, we prefer to plot the data as error bars showing standard deviations. We expand the “Statistics” section in the Methods to make this point clear.

To improve data visualization and avoid confusion, we removed dots/data points in plots of Variability and Efficacy.

3. Efficacy is a useful metric to use to compare each mutant/lesion population with its control, but it is a bit abstract and hard to imagine what that corresponds to for an individual larva's navigation behavior. In Figure 2, it would be helpful to plot mutant and het depth change vs. first bout direction as in Figure 1L to see the difference in slopes.

Thanks to this comment and similar comments from Reviewer 1, we revisited our original conception of “efficacy.” We updated the definition of efficacy so that it accounts for the total travel distance (Figures 1 L-O). Given the swim direction of a bout, a fish with higher depth change efficacy allocate more translocation to the vertical axis following consecutive bouts in the sequence (Figure 1L). We are grateful for the Reviewer’s critique.

We attempted the exact analysis proposed by Reviewer 3, but when we plotted the efficacy slope for every manipulation, but found it difficult to visualize two distributions on a scatter plot. Without the raw datapoints, the figure just looks like two fitted lines with different slopes. Instead, to help with understanding and visualization of this parameter, we included a schematic illustration for our definition of "depth change efficacy” in Figure 1L. We hope that the Reviewer finds this useful.

4. To further illustrate how gravity-blind larvae swim, I would like to see some representative trajectories of mutant and het fish, as in figure 1A, chosen from the mode of the distribution of efficacy in each population.

We truly appreciate this suggestion: On adding panel 2B, the figure does a much better job of illustrating the phenotype of *otog* -/- fish. To the second part of the comment, since efficacy is calculated using regression analysis on a large amount of swim sequences, it is not possible to pick trajectories with a certain efficacy (Figure 1 L-O).
