# Supplementary material for: Evolutionarily conserved brainstem architecture enables gravity-guided vertical navigation
Source: PLoS Biol. 2024 Nov 12;22(11):e3002902. doi: 10.1371/journal.pbio.3002902 (PMC11584107; doi:10.1371/journal.pbio.3002902)
Supplement: Attachment — Submitted filename: Response Final 241004.pdf [file pbio.3002902.s006.pdf]

Reviewer #1

My comments have been sufficiently addressed by the authors and I would be happy to see this paper published. I have a few small comments for the authors below.

We thank the reviewer for their careful review of our manuscript.

Minor issues with the text:

Line 63: "The INC/nMLF receives ascending inputs; lesions there disrupred heading and navigation efficacy." <- Lesions within the INC/nMLF or specifically of the ascending inputs?

We edited this sentence to specify lesions of the INC/nMLF.

Line 106: "A course of trajectory with greater direction changes results in higher veering." <- ?

To improve clarity, we changed this sentence to "A bout sequence with greater direction changes…"

Line 141: "In addition swim directions,..." <- "to swim directions"
Line 174: "... while lack the utricular..." <- "while they lack"
Line 244: "...to indicate the how strongly fish..." <- "to indicate how strongly the fish"

We fixed these typos.

Reviewer #2

It is worrying that the authors seem to think only interesting articles should be thoroughly reviewed. Now that the authors clarified the different experiments were done in different genetic backgrounds, the high variability makes more sense, it does show a wide range of natural WT behaviours which are all deemed effective by the authors, so I also appreciate the tempering of the claims regarding dramatic/crucial effects of their interventions. Overall, the authors have made all aspects of the manuscripts clearer and I am satisfied with their answers.

We are grateful that the reviewer's comments improved the clarity of our manuscript.

The added navigation figure for otog-/- is welcome and does show more random trajectories, and the impairment to depth navigation/changes appears a bit more clearly. I understand that there are biophysical constraints that limit the variability, my point was that if they were gravity blind, the veering would be more random, and so the variability higher. The article that the authors cite was not done in the dark, and the conclusion states that the small effects could be due to compensation strategies and training, so I am not sure it supports the small effects they observe. However, I am now reassured regarding the phenotype they observe.

We thank the reviewer for their thoughtful engagement and comments. We are pleased that the revisions to our manuscript have addressed their concerns about the navigation phenotype.

I appreciate the context and new discussion surrounding the VS lesions.

As the authors know, linear accelerometer alone cannot discriminate between tilt and translations, which the fish experience during each bout. As discussed in 10.1016/j.conb.2023.102776 zebrafish larvae may use the temporal dynamics of stimuli to discriminate between the two. And specificity was observed in the otolith organs in zebrafish (10.1038/s41467-022-35190-9), which could explain how the semicircular canals are not needed. But this may be out of the scope of the discussion in this manuscript.

The reviewer is correct that we are aware of these studies. It is likely that larvae rely on differences in the temporal dynamics to decode body tilts and linear acceleration. We agree that this topic falls outside the scope of the discussion in our manuscript.

Regarding other sensory modalities, and the claim that the fish only maintain trajectory is undermined by the efficacy of depth changes being only slightly affected in otog-/-. Although by their own arguments, it seems the efficacy is mostly an effect of motor circuits and not of gravity sensing. For the swim bladder, I was more thinking along the lines of what was observed in 10.1038/s41467-023-36682-y as a biomechanical factor in righting itself. But I appreciate that these would be outside of the scope of this manuscript.

We agree with the reviewer that, compared to the otolith-vestibulospinal circuit responsible for transforming gravity sensation into postural reflexes on the pitch axis, the otolith-TAN-nMLF/INC-spinal circuit is more directly involved in motor control.

The study the reviewer mentioned is already referenced in our manuscript: “the tangential-INC/nMLF circuit controls a vestibular-induced body bend reflex that allows fish to maintain posture on the roll (barbecue) axis.” We did not expand on this because addressing how torque on the roll axis contributes to posture and locomotion in the vertical plane is complex. We appreciate the reviewer's understanding that a discussion of biomechanical mechanisms is beyond the scope of our short report.
